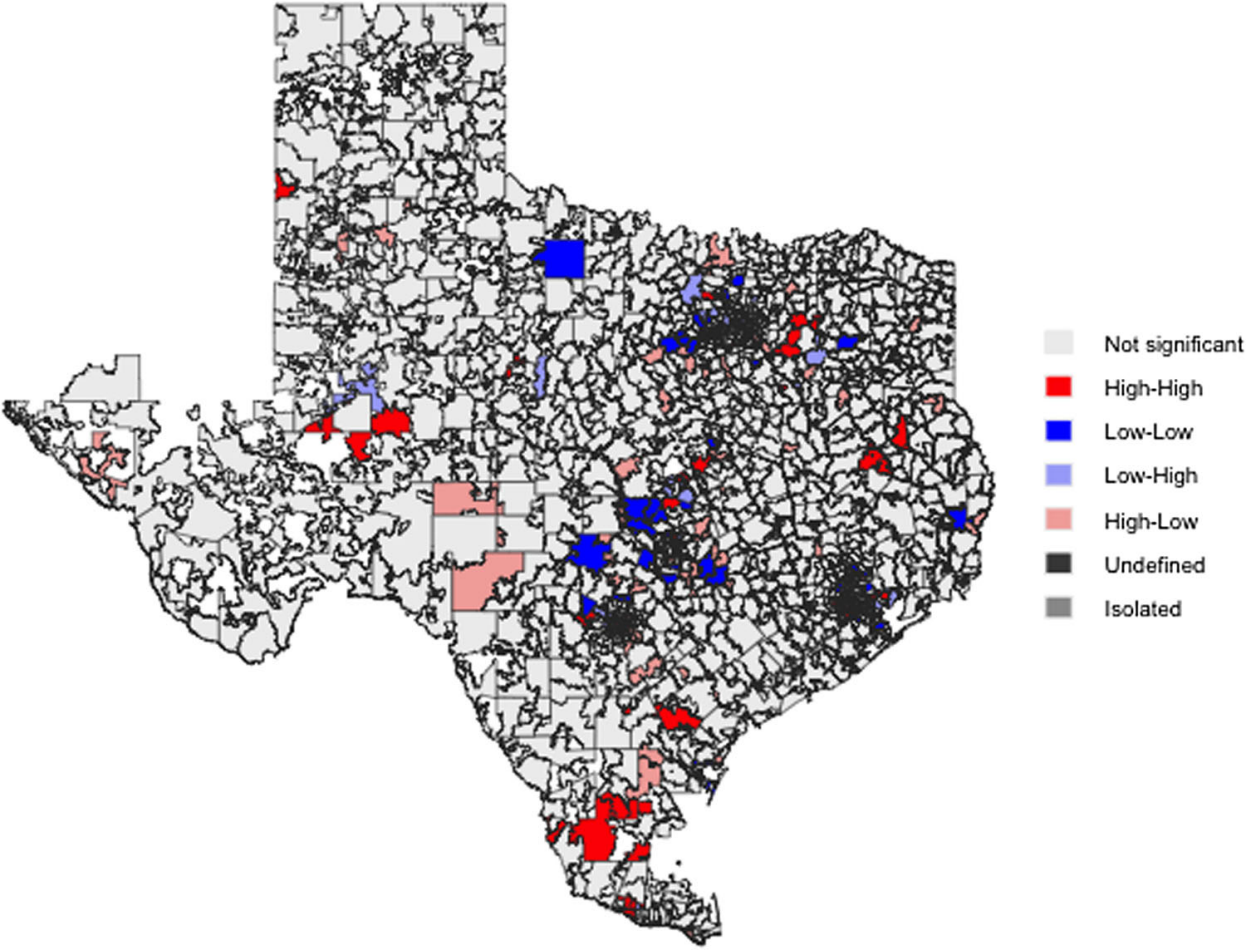# Analyzing the Relationship Between Socioeconomic Deprivation and Outpatient Medicare Part D Fluroquinolone Claims in Texas

**DOI:** 10.1017/ash.2024.205

**Published:** 2024-09-16

**Authors:** Mayar Al Mohajer, Edgar Samarasundera, Judite Gonçalves, Alicia Heath

**Affiliations:** Baylor College of Medicine; School of Public Health, Imperial College London; Imperial College London

## Abstract

**Background:** Only a few studies have assessed the relationship between deprivation and excessive antibiotic use. In Texas, antimicrobial prescription is particularly high compared with the rest of the US. This study analyzed the association between local area socioeconomic deprivation and providers’ fluoroquinolone claim rates among beneficiaries 65 years and older in Texas. **Method:** This ecological study utilized provider- and area-level data from Medicare Part D Prescribers and the Social Deprivation Index (SDI) repositories. To identify geographic patterns and autocorrelation in and between SDI and fluoroquinolone claims, spatial dependence of these two variables was assessed by bivariate Local Indicators of Spatial Association (LISA) cluster mapping along with the global and local Moran’s I analyses. Negative binomial regression models were employed to evaluate the relationship between provider- and area-level characteristics (prescriber’s gender, specialty, rural-urban community area, beneficiaries' demographics, area-level population, and normalized SDI) and fluoroquinolone claim rates per 1,000 beneficiaries. **Result:** A total of 11,996 providers were included. There was no spatial dependence between SDI and rates of fluoroquinolone claims in Texas (Global Moran’s I =0.01, P=0.618). Bivariant LISA maps showed 85 high-high and 38 low-low spatial clusters. Higher SDI (incidence rate ratio (IRR) 0.98, 95% confidence interval (CI) 0.97-0.99 per 1-unit increment) and male providers (IRR 0.96, 95%CI 0.94-0.99) were associated with lower claim rates. In contrast, several factors were associated with higher claim rates, including non-metropolitan areas (1.04, 95%CI 1.00-1.09), and practices with a high proportion of male patients (IRR 1.12, 95%CI 1.10-1.14), Black patients (IRR 1.05, 95%CI 1.03-1.07), or Medicaid beneficiaries (IRR 1.15, 95%CI 1.12-1.17). Effect modification was observed between SDI and rurality, with higher SDI in non-metropolitan areas associated with higher claim rates, whereas SDI in metropolitan areas was inversely related to claim rates. **Conclusion:** This study showed that the distribution of high and low SDI and rates of fluoroquinolone claims were more geographically clustered than expected by random chance alone. Lower fluoroquinolone claim rates among Texas Medicare providers were seen in metropolitan areas with higher SDI, indicating potential barriers to care. Conversely, higher claim rates were observed in rural areas with higher SDI, signifying a possible knowledge or attitude gap towards fluoroquinolone use. These findings provide opportunities for public health professionals to explore gaps in the knowledge and attitudes of patients and providers related to antimicrobial use, particularly in rural regions, and investigate barriers to healthcare access in metropolitan areas.

**Figure:**